# Case Report: Acute cerebral infarction as the initial manifestation of malignant tumors with trousseau syndrome in the elderly

**DOI:** 10.3389/fonc.2023.1188998

**Published:** 2023-11-29

**Authors:** Chen Li, Miao Fan, Wen He, Yingying Gong, Lei Su

**Affiliations:** ^1^ Department of Geriatrics, Sun Yat-sen University First Affiliated Hospital, Guangzhou, Guangdong, China; ^2^ Department of Medical Imaging, Sun Yat-sen University First Affiliated Hospital, Guangzhou, Guangdong, China

**Keywords:** Trousseau syndrome, acute cerebral infarction, malignant tumor, elderly patients, comprehensive geriatric assessment

## Abstract

Both acute cerebral infarction and malignant tumors are prevalent in the elderly. However, acute cerebral infarction is rarely present as the first clinical manifestation of malignant tumors. By searching the Picture Archiving and Communication System from 2010 to 2022 and the medical record database from 2003 to 2022, we found three cases of Trousseau syndrome, one male and two females with an average age of 69.3 ± 3.2 years, presenting with acute cerebral infarction. Two patients denied having hypertension, diabetes, and coronary heart disease. The average value of the D-dimer was 17.83 ± 12.39 mg/L (normal range, 0 to 0.55 mg/L). Magnetic resonance imaging (MRI) of the brain showed scattered and multiple small infarcts in the watershed area. The sites of infarction were not those that are typically caused by vascular atherosclerosis. One of the females was diagnosed with pancreatic cancer (T2N2M1, stage IV), the male was diagnosed with gastric cancer (T4N3M1, stage IV), and the other female was diagnosed with lung adenocarcinoma (rTxN3M1b, stage IV). The patient with pancreatic cancer underwent a comprehensive geriatric assessment, which revealed that she had a disability, dementia, malnutrition, short life expectancy, and high chemotherapy risk. Ultimately, the patient opted for conservative care, and 3 months after being discharged, she passed away from an acute upper gastrointestinal hemorrhage. Elderly patients with unexplained D-dimer elevation, multiple cerebral vascular lesions detected on MRI, and an absence of typical stroke risk factors need to be monitored for Trousseau syndrome. To screen for cancer, tumor markers and related imaging should be performed first. Trousseau syndrome is primarily treated with chemotherapy, radiotherapy and anticoagulant therapy. The risk of bleeding should be assessed carefully when using anticoagulant therapy in the elderly. Comprehensive geriatric assessment can assist in weighing the benefits and side effects of cancer treatment, making correct medical choices, and improving patients’ quality of life.

## Introduction

Cancer and thromboembolism are inextricably linked. Venous thromboembolism (VTE) affects roughly 15% of cancer patients, with nearly 50% showing evidence of VTE on postmortem examination (1). Arterial thromboembolic events account for 10–30% of thrombotic complications in cancer patients (2).

In 1865, Trousseau defined Trousseau syndrome as a thromboembolic complication caused by malignant tumors with a hyperactive coagulation state, including VTE, arterial thrombosis, disseminated intravascular coagulation, etc. (3). Acute cerebral infarction, one of the thromboembolic events, is rarely present as the first clinical manifestation of a malignant tumor.

**Figure 1:**
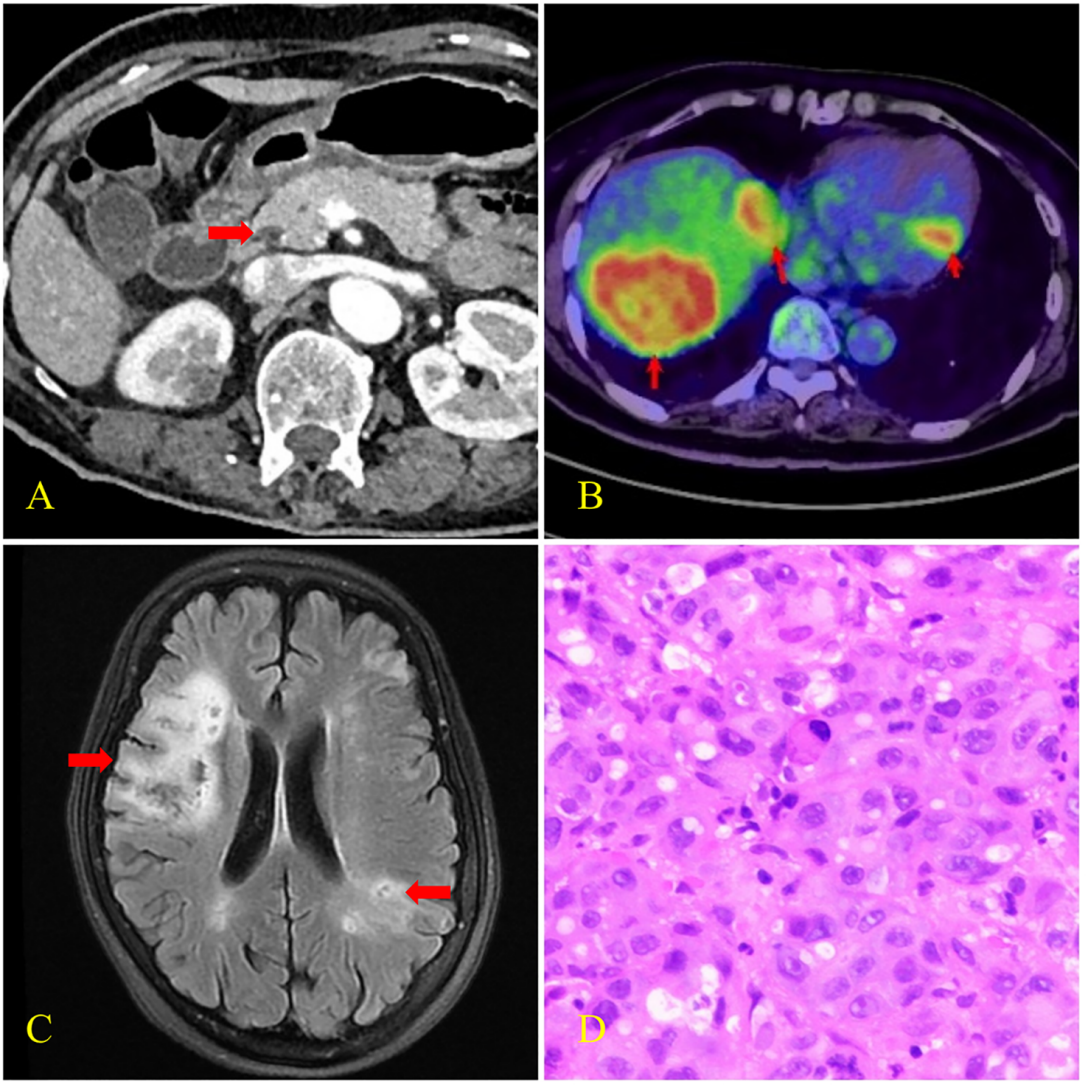
CT, PET-CT, brain MRI, and histopathological findings of case 1. **(A)** Enhanced CT scan revealed pancreatic head carcinoma (arrow) with the size of 14×11mm. **(B)** PET-CT scan revealed multiple liver metastases and left ventricular wall hypermetabolic foci (arrows) with SUVmax of 15.6 and 9.8 respectively. **(C)** Brain MRI revealed multiple focal points of ischemia, infarction, and softening (arrows). **(D)** Biopsy histology showed poorly differentiated adenocarcinoma infiltration in the liver puncture tissue.

**Figure 2:**
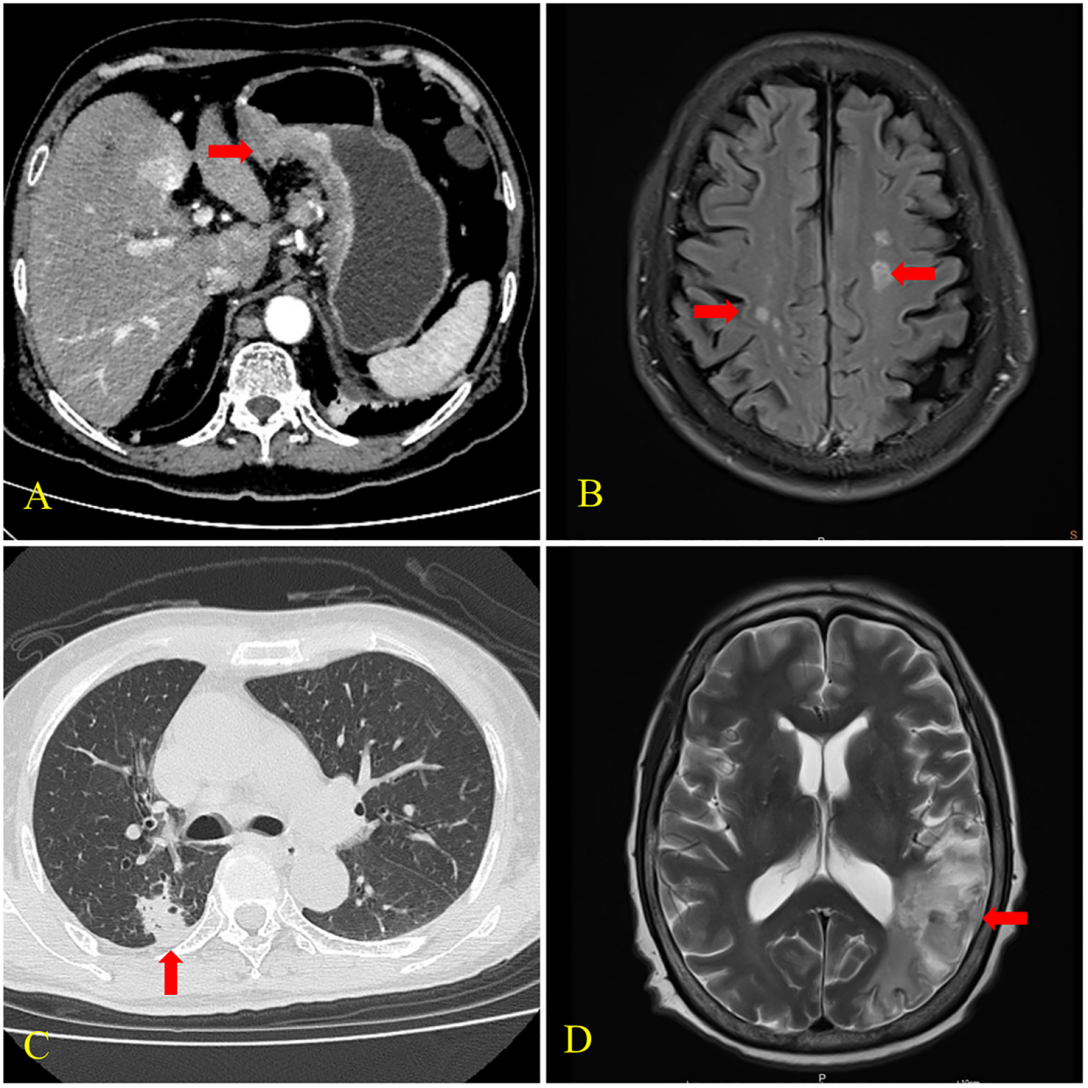
CT and brain MRI findings of cases 2 and 3. **(A)** CT scan of case 2 showed uneven thickening of the gastric wall and frizzy serosal surface (arrow). **(B)** MRI of case 2 revealed multiple cerebral infarctions in the bilateral fronto-parietal occipital lobe and central semiovale (arrows). **(C)** CT scan of case 3 showed a nodular shadow with the size of 18×16mm in the posterior segment of the upper lobe of the right lung (arrow). **(D)** MRI of case 3 showed cerebral infarctions in the right frontal lobe and left temporoparietal occipital lobe (arrow).

**Figure 3:**
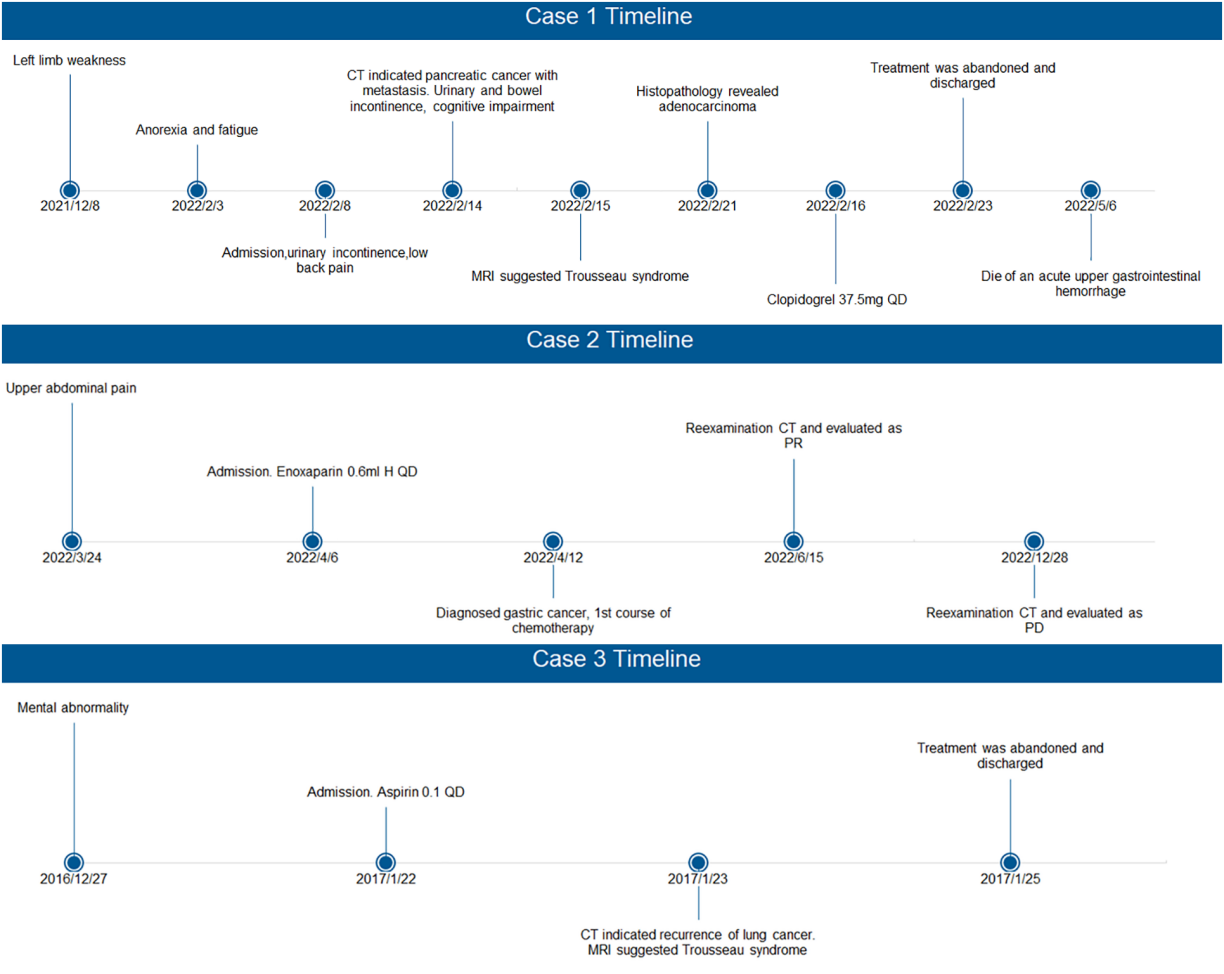
Timelines of the 3 cases. PR, partial response; PD, progressive disease.

In 2022, a 67-year-old female was admitted with acute cerebral infarction. She was diagnosed with Trousseau syndrome and pancreatic cancer after admission. By searching the Picture Archiving and Communication System (PACS) (2010–2022) using the keyword “Trousseau syndrome”, we identified two other cases. PACS is an image system in the hospital, not a database. The software mainly consists of medical image storage and control software, medical diagnosis and imaging system, image reading workstation and architecture service platform. The main task is to digitally store all kinds of medical images produced daily, and when necessary, it can be quickly recalled and used under certain authorization, and at the same time add some auxiliary diagnostic management functions. Since Trousseau syndrome has no code in the International Classification of Diseases (ICD), we used “paraneoplastic syndrome of the nervous system” to search the discharged medical records (2003–2022) and found 123 cases. One more patient with Trousseau syndrome was found after reviewing the MRI and medical history of these 123 cases. Due to inadequate clinical data, one outpatient was excluded. Thus, in the present study, we report three cases of Trousseau syndrome, presenting with acute cerebral infarction. Detailed clinical characteristics of these cases are shown in **Table 1**. Image data and histopathological findings are shown in **Figures 1**, **2**. Timelines of the 3 cases are shown in **Figure 3**.

**Table 1:** Detailed clinical characteristics of the three cases.

	Case 1	Case 2	Case 3
Gender	Female	Male	Female
Age	67	73	68
Date of admission	2022-02-08	2022-04-06	2017-01-22
Chief complaint	Weakness of the left limb, anorexia for 2 months, and fatigue for 5 days	Upper abdominal pain for half a month	Mental abnormality for 1 month
Blood pressure (mmHg)	121/95	120/82	135/73
Pulse rate (beats/min)	104	98	100
Previous history	Left lower extremity deep vein thrombosis	Hypertension	Lung cancer, left femur fracture after internal fixation
Physical examination of the nervous system	The right-hand grip reflex was positive. Left upper limb proximal muscle strength was grade 2, distal muscle strength was grade 3, and left lower limb muscle strength was grade 4. The Babinski sign was positive	N/A	Irritability and incoordination. The right palpebral fissure was slightly smaller and the left nasolabial fold was shallow. The Babinski sign was positive and the meningeal irritation sign was probably positive
CRP mg/L (0.00–10.00)	162.1	11.04	8
AFP μg/L (0.00–20.00)	2.23	3.97	7.58
CEA μg/L (0.00–5.00)	72.01	20.85	38.42
CA19-9 U/mL (0.00–35.00)	>12000	19.15	33.73
CA125 U/mL (0.00–35.00)	3990.7	105.1	13.03
D-dimer mg/L (0.00–0.55)	28.59	20.61	4.28
PT s (11.0–14.0)	13.5	14	12.4
APTT s (25.0–31.2)	30.4	32.5	24.6
INR (0.80–1.15)	1.24	1.27	1.04
Fibrinogen g/L (1.80–3.50)	3.38	1.13	2.04
TAT ng/mL (0.0–4.0)	34.3	N/A	N/A
TM TU/mL (3.8–13.3)	14.9	N/A	N/A
TnT-T ng/mL (0.000–0.014)	0.305	0.088	N/A
CK-MB ng/mL (0.10–4.94)	5.89	0.93	N/A
CHOL mmol/L (3.1–5.7)	4.0	4	5.3
LDL-C mmol/L (0.33–1.70)	2.61	2.58	3.24
ALT U/L (1–40)	33	13	9
AST U/L (1–37)	51	20	22
Bilirubin μmol/L (0.5–7.0)	8.1	12.9	13.3
Echocardiography	Aortic insufficiency (mild)	Aortic and left atrial enlargement, aortic insufficiency (mild), mitral insufficiency (mild-moderate), decreased diastolic function	Pericardial effusion (small amount)
Vascular ultrasound of the lower extremity	Deep vein thrombosis on both sides	No thrombosis	N/A
MRI	Multiple focal points of ischemia, infarction, and softening, some of which were acute	Multiple cerebral infarctions in the bilateral fronto-parietal occipital lobe, central semiovale, and left occipital lobes, most of which were subacute	Cerebral infarctions in the right frontal lobe and left temporoparietal lobe. Ischemic focus in the right cerebellum and bilateral frontoparietal lobe
Histopathology	Poorly differentiated adenocarcinoma infiltration in the liver puncture tissue, considering the origin of the pancreaticobiliary duct	Poorly differentiated gastric adenocarcinoma	N/A
Immunohistochemical results	MUC-1(+), MUC-5AC(+), MUC-2(-)	CK(+), CerbB2(2+), M-CEA partly(+)	N/A
Diagnosis	Pancreatic cancer (T2N2M1, stage IV) and Trousseau syndrome	Gastric cancer (cT4N3M1, stage IV) and Trousseau syndrome	Lung adenocarcinoma (rTxN3M1b, stage IV) and Trousseau syndrome
Anticoagulant/antiplatelet therapy	Plavix 37.5mg QD	Enoxaparin 0.6ml H QD	Aspirin 0.1 QD
Chemotherapy	N/A	Course 1: Oxaliplatin+S-1 Courses 2–6: Oxaliplatin+S-1+Herceptin+ Keytruda	N/A
Prognosis	Died of gastrointestinal bleeding 3 months later	Under regular chemotherapy and still alive until 2022-12-28	N/A

CRP, c-reactive protein; AFP, alpha-fetoprotein; CEA, carcinoembryonic antigen; CA19-9, carbohydrate antigen 19-9; CA125, carbohydrate antigen 125; PT, Prothrombin time; APTT, activated partial thromboplastin time; INR, international normalized ratio; TAT, thrombin–antithrombin complex; TM, thrombomodulin; TnT-T, troponin T; CK-MB, creatine kinase-MB; CHOL, cholesterol; LDL-C, low-density lipoprotein cholesterol; ALT, alanine aminotransferase; AST, aspartate transaminase; MRI, magnetic resonance imaging; N/A, not available.

## Case reports

### Case 1

A 67-year-old female was hospitalized in 2022 due to weakness in her left limb, anorexia, and fatigue. Brain Magnetic Resonance Imaging (MRI) performed at the local hospital prior to admission revealed small acute hemorrhages in the right frontal white matter area and numerous infarcts in the bilateral frontal and parietal cortex, left temporal cortex and white matter area, and right cerebellar hemisphere. Multiple hepatic space-occupying lesions were detected using abdominal ultrasound. She had been diagnosed with VTE owing to swelling and pain in her left lower limb 1 month before admission.

Physical evaluation revealed that the proximal muscle strength of the left upper limb was grade 2, the distal muscle strength was grade 3, and the muscle strength of the left lower limb was grade 4. The Babinski sign was positive. The serum carbohydrate antigen 19-9 (CA19-9) level was >12000.00 U/mL (normal range: 0 to 35 U/mL), serum carcinoembryonic antigen (CEA) level was 72.01 ug/L (normal range: 0 to 5μg/L), and serum carbohydrate antigen 125 (CA125) level was 3990.70 U/mL (normal range, 0 to 35 U/mL). D-dimer was 28.59 mg/L (normal range: 0 to 0.55 mg/L), thrombomodulin (TM) was 14.9 TU/ml (normal range: 3.8 to 13.3 TU/ml), and serum thrombin–antithrombin complex (TAT) was 34.3 ng/ml (normal range: 0 to 4 ng/ml). High-sensitivity troponin T (TNT-T) was 0.305 ng/ml (normal range: 0.000–0.014 ng/ml). Vascular ultrasonography of the lower extremity revealed deep venous thrombosis. Positron emission tomography–computed tomography (PET–CT) scans showed pancreatic head carcinoma with multiple metastases of the liver, kidney, and lymph nodes. MRI of the brain showed multiple focal points of ischemia, infarction, and softening. Liver histopathology revealed poorly differentiated adenocarcinoma infiltration. Immunohistochemistry revealed positive Mucin-1 and Mucin-5AC and negative Mucin-2. The patient was diagnosed with pancreatic cancer (T2N2M1, stage IV) and Trousseau syndrome.

According to the Clinical Frailty Scale (CFS), the patient was rated as category 7, severely frail. We performed a comprehensive geriatric assessment (CGA) for the patient. The Barthel index indicated severe impairment in the ability to perform daily living (ADL scored 10). The possibility of falls was significant (The Johns Hopkins Fall Risk Assessment Scale scored 7). She had mild dementia. The Mini-Mental State Examination (MMSE) scored 12. She had mild depression. The short version of the Geriatric Depression Scale (GDS-15) scored 8. She was at risk of malnutrition. The Mini-Nutritional Assessment (MNA) scored 7. The Padua assessment model score was 7, indicating that she was at risk of lower extremity venous thrombosis. She was also at risk of bleeding because the IMPROVE Bleeding Risk Score was 3. The grade for the water swallow test was 2. The performance percentage assessed by the Karnofsky Performance Scale (KPS) was 30%, indicating poor treatment response and chemotherapy tolerance. Grade 3 on the Zubrod-ECOG-WHO scale indicated that the patient was unfit for chemotherapy. The ePrognosis score was 14 with an estimated 1-year mortality risk of 64%. The result of the Chemo-toxicity Calculator predicted an increased risk of chemotherapy (the score was 11 and the predictive value was 78%).

The patient was prescribed clopidogrel 37.5mg QD, butylphthalide 25mg iv drip QD, and other medications including anti-depressants, antibiotics, etc. She was discharged to home care at her family’s request; three months later, she passed away from an acute upper gastrointestinal hemorrhage.

### Case 2

A 73-year-old male presented with “upper abdominal pain for two weeks” was hospitalized in 2022. He had cerebral infarction half a month before and a 10-year history of hypertension. He showed no evidence of positive neurological signs. Computed tomography (CT) scans showed numerous lymph node metastases in addition to gastric cancer. Gastroscopy and biopsy histopathology showed a poorly differentiated gastric adenocarcinoma. CK (+), CerbB2 (2+), and M-CEA (+) partly were revealed on immunohistochemistry. MRI of the brain revealed multiple infarctions in the bilateral fronto-parietal occipital lobe and central semiovale. The patient was diagnosed with gastric cancer (cT4N3M1 stage IV) and Trousseau syndrome. He was treated with 0.6ml of enoxaparin injected subcutaneously daily. Meanwhile, chemotherapy was started, and two months later, a CT scan revealed that the tumor was in partial remission.

### Case 3

A 68-year-old female was hospitalized due to “mental behavior abnormalities for one month” in 2017. Her clinical manifestations were raving, hallucinations, and beating and swearing at others. She had a history of lung adenocarcinoma after surgery and chemotherapy for nearly 3 years. Her right palpebral fissure was slightly smaller on physical examination, her left nasolabial fold was shallow, and the Babinski sign was positive. A chest CT scan showed a nodular shadow in the posterior segment of the upper lobe of the right lung, multiple lymph node metastases, and a soft tissue mass shadow in the right anterior chest wall. MRI of the brain revealed infarctions in the right frontal lobe and left temporoparietal occipital lobe and ischemic focus in the right cerebellum and bilateral frontoparietal lobe. The patient was diagnosed with recurrent lung adenocarcinoma (rTxN3M1b stage IV) and Trousseau syndrome. She received aspirin 0.1g daily and was discharged after her mental symptoms abated. Unfortunately, she lost follow up.

## Discussion

The three cases diagnosed with Trousseau syndrome shared some common features: 1. They were all elderly over 60 years old. 2. The primary disease for all was adenocarcinoma. 3. They all showed cerebral infarction. MRI of the brain showed scattered and multiple small infarcts in the watershed area and those sites were not the typical ones that are caused by vascular atherosclerosis. 4. None had typical risk factors for stroke such as diabetes, coronary heart disease, and long-term smoking. Case 2 had a history of hypertension, but his blood pressure was normal during the physical examination. 5. Unexplained D-dimer elevation. In some cases, lower extremity deep vein thrombosis can be detected.

There is no ICD code for Trousseau syndrome, which makes it harder for clinicians to diagnose the condition and reduces the acuity of early judgment.

The possible pathogenesis of Trousseau syndrome includes the following (2, 4–6). 1. Hypercoagulability caused by tissue factors, cysteine proteases, plasminogen activators, plasminogen activator inhibitors, inflammatory cytokines, tumor necrosis factors, and interleukin and mucin expressed or secreted by tumor cells. 2. Procoagulant substances released due to the interaction of tumor cells, vascular endothelial cells, platelets, and mononuclear macrophages. 3. Tumor-induced hypoxic environment. 4. Adenocarcinoma, which may secrete mucin, can promote platelet aggregation and lead to thromboembolism; for example, in case 1, Mucin-1 and Mucin-5AC were positive. The most common malignancies associated with Trousseau syndrome are mucinous carcinoma of the pancreas or gastrointestinal tract, lung cancer, and ovarian cancer (7).

Acute cerebral infarction, which may be caused by intravascular coagulation and cardiac embolism, is rarely present as the first manifestation of cancer. The sites of infarction associated with Trousseau syndrome detected on MRI were not those that are typically caused by vascular atherosclerosis. Bao et al. divided cerebral infarction associated with Trousseau syndrome into three types: 1. Single lesion (Type 1). 2. Scattered lesions in one vascular area (Type 2). 3. Multiple lesions in multiple vascular regions (Type 3), including unilateral anterior circulation (Type 3A), posterior circulation (Type 3B), bilateral anterior circulation (Type 3C), and both anterior and posterior circulations (Type 3D) (8). The cases we report are all type 3, the most prevalent.

Currently, there is no accepted diagnostic criterion for Trousseau syndrome. Tsushima et al. found that elevated D-dimer and CRP levels in patients with cerebral embolism may have diagnostic values for Trousseau syndrome, with cutoff values of 2.68 g/mL fibrinogen equivalent units and 0.29 mg/dL, respectively (9). All the cases we report meet this diagnostic criterion.

The primary treatment for Trousseau syndrome is tumor burden reduction and anticoagulation (1, 7). However, when patients are diagnosed, they are usually in an advanced stage, and tumor reduction therapy is difficult to implement. First and foremost, anticoagulation should be performed, with low-molecular-weight heparin being the preferred medication (4). New oral anticoagulants may be explored if long-term injection therapy is not tolerated (10). It is worth noting that elderly patients, as in Case 1, are at a higher risk of bleeding when using anticoagulants or antiplatelet agents. The risk of thrombosis and bleeding should be weighed carefully.

Cancer is more common in the elderly, and cerebral infarction with cancer has a poor prognosis. Kneihsl et al. discovered that patients hospitalized for ischemic stroke with “active cancer” had a substantially higher mortality rate than those with “non-active cancer” (11). Treatment tolerance is also lower in the elderly. CGA is a systematic assessment process that includes eight geriatric domains (physical performance, functional status, comorbidity, polypharmacy, cognition, nutrition, social support, and psychological status) (12). CGA of case 1 showed that she had a disability, dementia, malnutrition, short life expectancy, and high chemotherapy risk. Her wishes were respected and chemotherapy was avoided in the final medical decision. CGA can aid in weighing the benefits and side effects of chemotherapy, aiding in the formulation of proper medical judgments, and assisting patients in enhancing their quality of life.

## Conclusion

Elderly patients with unexplained D-dimer elevation, multiple cerebral vascular lesions detected on MRI, and an absence of typical stroke risk factors need to be monitored for the likelihood of Trousseau syndrome. To screen for cancer, tumor markers and related imaging should be performed. The primary treatment options for Trousseau syndrome are tumor burden reduction and anticoagulant therapy, and the risk of bleeding should be carefully evaluated when anticoagulant drugs are used in the elderly. CGA can aid in weighing the benefits and side effects of cancer treatment, aiding in the formulation of proper medical judgments, and assisting patients in enhancing their quality of life.

## Data availability statement

The original contributions presented in the study are included in the article/supplementary material. Further inquiries can be directed to the corresponding author.

## Ethics statement

Written informed consent was obtained from the individual(s) for the publication of any potentially identifiable images or data included in this article.

## Author contributions

LS, CL, WH and YG contributed to the implement of the treatment. CL and MF contributed to the collection, analysis and interpretation of data, drafting and revision of the manuscript. LS contributed to the conception of the treatment, revision and approval of the final manuscript. All authors approved the submitted version.
